# Pharmacokinetics and Anti-Tumor Efficacy of PEGylated Liposomes Co-Loaded with Cisplatin and Mifepristone

**DOI:** 10.3390/ph16101337

**Published:** 2023-09-22

**Authors:** Karen Ocaña-Arakachi, Julio Martínez-Herculano, Rafael Jurado, Monserrat Llaguno-Munive, Patricia Garcia-Lopez

**Affiliations:** 1 Laboratorio de Fármaco-Oncología, Subdirección de Investigación Básica, Instituto Nacional de Cancerología, Mexico City 14080, Mexico; arakachi_karendanahi@outlook.com (K.O.-A.); julenri_reiko@hotmail.com (J.M.-H.); fcojl@yahoo.com (R.J.); muniv1250@hotmail.com (M.L.-M.); 2 Posgrado en Ciencias Biológicas, Universidad Nacional Autónoma de México, Mexico City 04510, Mexico; 3 Laboratorio de Física Médica, Subdirección de Investigación Básica, Instituto Nacional de Cancerología, Mexico City 14080, Mexico

**Keywords:** bioavailability, cisplatin, co-encapsulation, liposomes, mifepristone, pharmacokinetics, synergism

## Abstract

Although cisplatin is an effective chemotherapy drug used against many types of cancer, it has poor bioavailability, produces severe adverse effects, and frequently leads to tumor resistance. Consequently, more effective formulations are needed. The co-administration of cisplatin with mifepristone, which counters an efflux pump drug-resistance mechanism in tumor cells, has shown important synergism, but without resolving the problem of poor bioavailability. Specificity to tumor tissue and bioavailability have been improved by co-encapsulating cisplatin and mifepristone in a liposomal formulation (L-Cis/MF), which needs further research to complete pre-clinical requirements. The aim of this current contribution was to conduct a pharmacokinetic study of cisplatin and mifepristone in male Wistar rats after administration of L-Cis/MF and the conventional (unencapsulated) formulation. Additionally, the capacity of L-Cis/MF to reduce tumor growth in male nude mice was evaluated following the implantation of xenografts of non-small-cell lung cancer. The better pharmacokinetics (higher plasma concentration) of cisplatin and mifepristone when injected in the liposomal versus the conventional formulation correlated with greater efficacy in controlling tumor growth. Future research on L-Cis/MF will characterize its molecular mechanisms and apply it to other types of cancer affected by the synergism of cisplatin and mifepristone.

## 1. Introduction

Cisplatin is one of the most active anti-cancer drugs currently used in chemotherapy; it is applied to more than 50% of human cancers, including lung, head and neck, colon, bladder, ovarian, and cervical cancer. However, its lack of specificity to tumoral tissue leads to dose-dependent damage to normal tissue. Consequently, the administration of cisplatin is associated with significant adverse effects such as nephrotoxicity, neurotoxicity, ototoxicity, and myelotoxicity [1,2]. In addition to these adverse effects, another disadvantage of cisplatin treatment is the rapid development of resistance; thus, most patients experience therapy failure and relapse, resulting in a poor prognosis. Therefore, combining cisplatin with other agents has been investigated to improve the therapeutic response.

A common strategy in combination chemotherapy is the inclusion of a sensitizing agent, which has no direct cytotoxic effect on cancer cells but can improve the efficacy of the antineoplastic drug [3,4,5]. According to the evidence in the literature, one of the main mechanisms of action for a chemotherapy sensitizing agent is the inhibition of a drug-transport protein known as permeability glycoprotein (P-glycoprotein or P-gp) [6]. Since P-gp acts as an efflux pump in cells, its inhibition undermines the mechanism of multi-drug resistance. Many anticancer drugs, including cisplatin, are P-gp substrates. Hence, the administration of cisplatin with a P-gp inhibitor should increase its effectiveness.

Some groups have investigated treatments combining a cytotoxic drug with an antihormonal agent such as raloxifene, tamoxifen, or medroxyprogesterone [7]. However, the doses of these combinations in conventional formulations have not produced selectivity for tumor tissue. As a consequence, adverse effects have been triggered by the treatment schemes tested. Other research groups have demonstrated the efficacy of mifepristone (RU-486, an antiprogestin drug) as a chemosensitizer for improving cisplatin efficacy. In the last few years, our group has researched the effect of the antiprogestogen mifepristone as a possible sensitizing agent for chemotherapy with cisplatin, finding that it increases therapeutic efficacy [8,9]. Indeed, the addition of mifepristone to cisplatin produces an important synergism in chemo-radiotherapy for cervical cancer cells as well as cervix xenografts [8].

Despite the synergism between cisplatin and mifepristone, the chemical instability of cisplatin leads to limited bioavailability. That is, it does not reach an adequate concentration at the tumor site [10,11]. The biodistribution of cisplatin is limited by its binding to plasma proteins, which irreversibly inactivates more than 90% of the drug, leaving only a small percentage of the dose for cytotoxic activity. Additionally, there is evidence that tubular nephrotoxicity is related to the pharmacokinetic parameters of plasma cisplatin [12,13]. On the other hand, mifepristone is administered orally with an absorption rate of approximately 70% from the gut. Unfortunately, it undergoes a first-pass effect in the liver, which diminishes its bioavailability to 40% [14].

An improvement in the bioavailability of cancer chemotherapy agents has been achieved with the development of liposomal drug carriers, which provide increased activity and reduced side effects. Our group recently reported the development of a liposomal system to co-deliver cisplatin and mifepristone. After preparing the liposomes with a reverse-phase evaporation method, the physicochemical characterization revealed a particle size of around 109 nm, a uniform dispersion of particles with a polydispersity index (PDI) of 0.11, and a Zeta potential (mV) of −38. Drug release profiles (at room temperature) exhibited a maximum release of 15% for cisplatin and ~60% for mifepristone at 96 h, showing good stability in the formulation. The co-encapsulation efficiency was 18% for cisplatin and 25% for mifepristone [15].

The delivery of cisplatin and mifepristone by the liposomal formulation (denominated L-Cis/MF) produced notable cytotoxic activity against cervical cancer cells, an apoptotic effect in the same cells, and a significant decrease in tumor growth in mice with a xenotransplant of HeweLa cells La cells [15]. Hence, it is important to continue with the preclinical characterization of L-Cis/MF

The aim of the current contribution was to carry out a pharmacokinetic study of cisplatin and mifepristone in male Wistar rats post-administration of L-Cis/MF and the conventional (unencapsulated) formulation. Additionally, an evaluation was made of the capacity of L-Cis/MF (and the conventional formulation) to reduce tumor growth in male nude mice after implanting xenografts of non-small-cell lung cancer.

## 2. Results

The preparation of the liposomal formulation that co-encapsulated cisplatin and mifepristone (L-Cis/MF) was prepared and characterized as mentioned in our previous work [15]. We realized the characterization of the batch of L-Cis/MF used in this work and the results were a particle size of 116.8 nm, a polydispersity index (PDI) of 0.17, and a Zeta potential (mV) of −36. The co-encapsulation efficiency was 22% for cisplatin and 22% for mifepristone.

### 2.1. In Vitro Cisplatin and Mifepristone Release from L-Cis/MF

**Figure 1:**
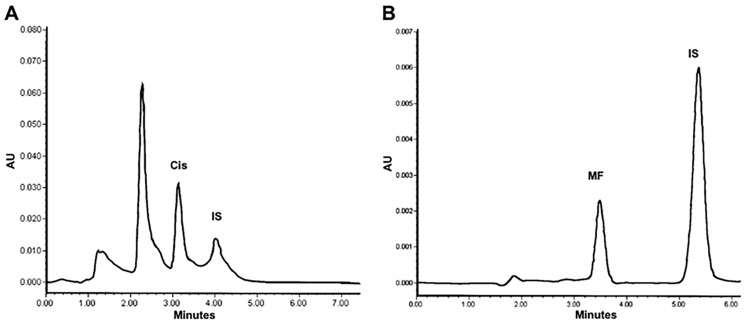
Chromatograms of liposomal cisplatin: (**A**) blank liposomes spiked with 5 μg/mL of cisplatin (Cis) and 50 μg/mL of nickel chloride, the internal standard (IS); (**B**) blank liposomes spiked with 1 μg/mL of mifepristone (MF) and 1 μg/mL of promegestone, the internal standard (IS).

To quantify the cisplatin and mifepristone released from L-Cis/MF, the amount of each drug found by HPLC was compared to calibration curves with known concentrations. The typical chromatograms obtained from the cisplatin standard solution spiked in blank liposomes show a retention time of 3.1 min for cisplatin and 4.0 min for nickel chloride, its internal standard (Figure 1A). The typical chromatograms of the mifepristone standard solution spiked in blank liposomes reveal a retention time of 3.4 min for mifepristone and 5.2 min for promegestone, its internal standard (Figure 1B).

**Figure 2:**
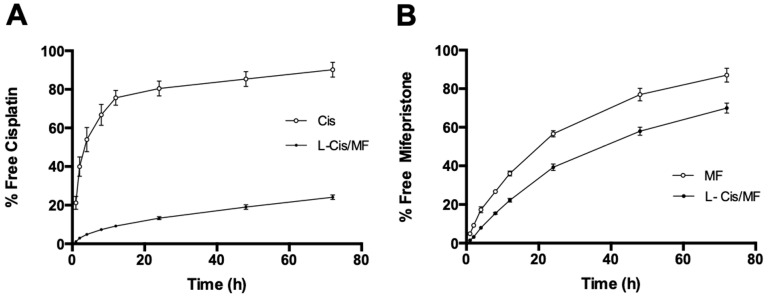
The percentage of cisplatin (**A**) and mifepristone (**B**) released from the L-Cis/MF formulation during 72 h of incubation at 37 °C in human plasma. Values are expressed as the mean ± SEM (n = 3).

Drug-release profiles of L-Cis/MF in human plasma (Figure 2) show a maximum release of 24% for cisplatin (Figure 2A) and 70% for mifepristone, both at the final reading (72 h) (Figure 2B).

The release profile is an important parameter that needs to be measured to evaluate the stability of the nanosystem. Drug release occurs as a function of membrane stability, so the drug release from liposomes can be attributed to the diffusion of the drug through the lipid layer and the disassembly of liposomes caused by various factors like the medium, pH, and temperature, among others [16]. The slow release of cisplatin from the liposome can be attributed to the rigidity of the liposomal membrane and the physicochemical characteristics of cisplatin. In contrast, the rate of mifepristone release from liposomes was the highest. The composition of the lipidic membrane and the lipophilicity of mifepristone strongly influence the dynamics of drug release. Furthermore, in vivo drug release from liposomes depends on several factors, like the interaction between lipid membranes with enzymatic degradation. Additionally, in vitro release experiments could not accurately predict the in vivo drug release. Therefore, pharmacokinetic and efficacy studies are necessary.

### 2.2. Pharmacokinetics of L-Cis/MF

**Figure 3:**
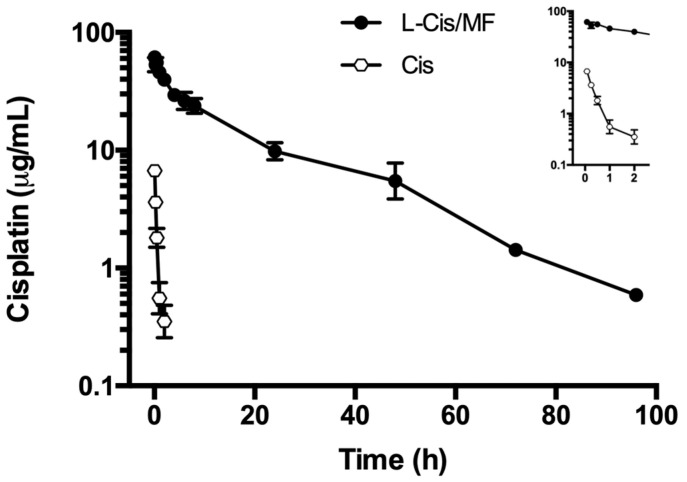
The plasma concentration of cisplatin in rats during the 96 h after the intravenous administration of cisplatin solution (Cis) or cisplatin-loaded liposomes (L-Cis/MF). In each case, the dose of cisplatin was 6 mg/kg. Each point represents the mean ± SEM (n = 6).

The plasma concentration of cisplatin in the animals under study (Figure 3) was significantly higher post-administration of L-Cis/MF, versus the conventional (unencapsulated) formulation of cisplatin (both injected IV). The elevated plasma concentration of cisplatin generated by the L-Cis/MF treatment declined slowly and steadily but remained relatively high at 48 h. Contrarily, the plasma concentration of cisplatin derived from the conventional formulation dropped rapidly during the first 2 h. After analyzing the plasma concentration of cisplatin over time for the liposomal and conventional formulations, the results were fitted to a two-compartment model.

**Figure 4:**
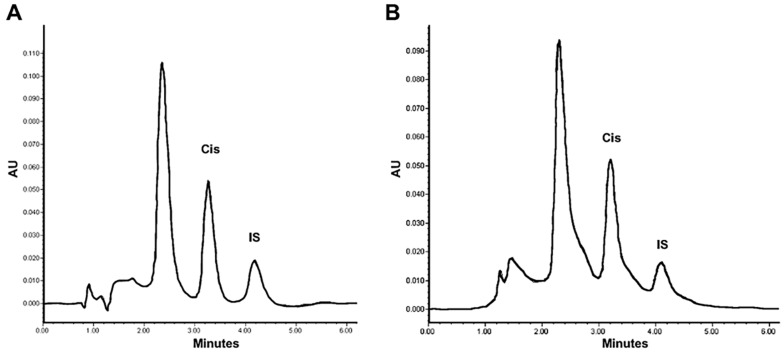
Chromatograms of cisplatin in plasma from blood samples drawn from rats: (**A**) at 5 min post-injection of 6 mg/kg of conventional cisplatin, and (**B**) at 1 h post-injection of 6 mg/kg of liposomal cisplatin; the samples were diluted 1:10 (**B**) and spiked with 50 μg/mL of internal standard (IS).

The corresponding pharmacokinetic parameters are listed in Table 1. The bioavailability of liposomal cisplatin, represented by AUC, was over 152-fold greater than that of conventional cisplatin. Furthermore, the Cmax and t_½_ were approximately 6-fold and 3-fold higher, respectively, for liposomal versus conventional cisplatin; while the Vd and Cl were about 4-fold and 162-fold lower, respectively (Table 1). Figure 4 portrays typical chromatograms of cisplatin in plasma from blood samples drawn from the rats, one at 5 min post-injection of 6 mg/kg of conventional cisplatin and the other at 24 h post-injection of 6 mg/kg of liposomal cisplatin (L-Cis/MF), the latter diluted 1:10.

**Figure 5:**
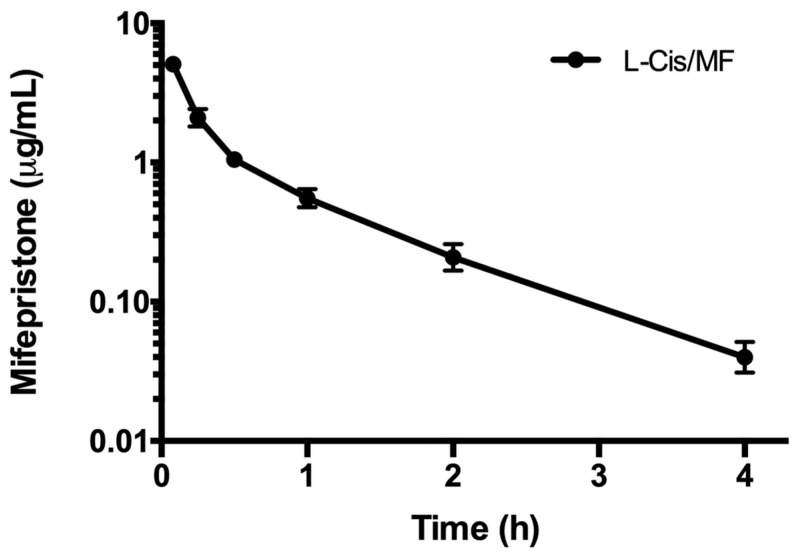
The plasma concentration of mifepristone in rats during the 4 h after intravenous injection of mifepristone-loaded liposomes (L-Cis/MF). The dose was 6.17 mg/kg of mifepristone. Each point represents the mean ± SEM (n = 6).

**Figure 6:**
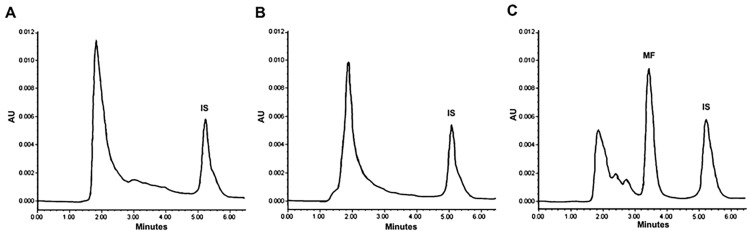
Chromatograms of the concentration of mifepristone in plasma from blood samples drawn from rats. Mifepristone was undetectable in samples (spiked with promegestone, the internal standard) taken at 5 min (**A**) and 1 h (**B**) after SC administration of a dose of 6.17 mg/kg of free mifepristone. Contrarily, mifepristone is clearly observed in a representative chromatogram of plasma from a blood sample drawn from a rat at 1 h (**C**) after a single intravenous injection of the same dose of mifepristone in the liposomal formulation (L-Cis/MF).

The pharmacokinetics of mifepristone were determined after administering the conventional and liposomal formulations (Figure 5). Analysis of the time course of the plasma concentration of mifepristone derived from the liposomal formulation was fitted to a two-compartment model. Whereas mifepristone was found in plasma for up to 4 h after the IV administration of L-Cis/MF, it was not detected at any sampling time post-injection of the conventional formulation (Figure 6). Since mifepristone administered conventionally was below the concentration quantifiable by the method of measurement used (0.05 μg/mL), the pharmacokinetic parameters of free mifepristone could not be determined (Table 2).

### 2.3. Tumor Growth Inhibition and Systemic Toxicity

**Figure 7:**
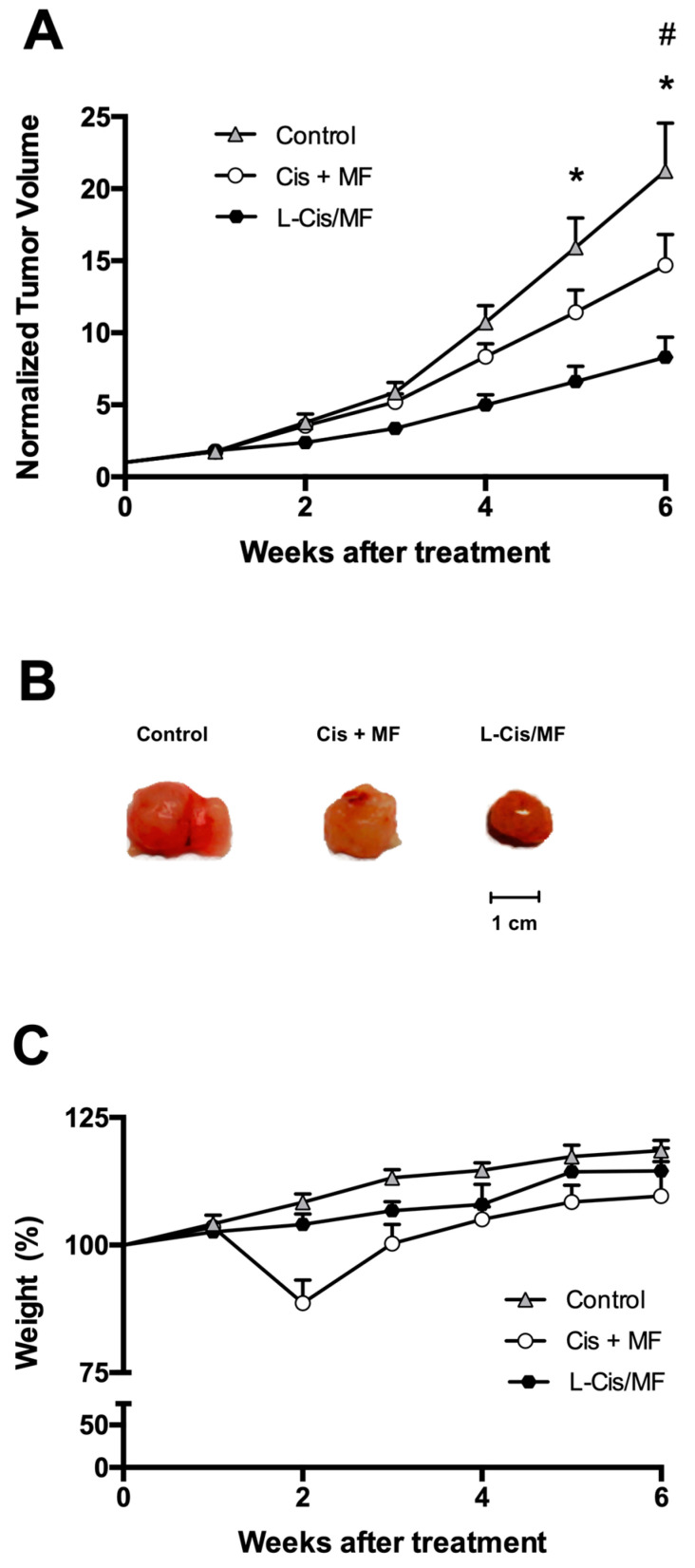
Efficacy of the 3-week treatments after subcutaneously implanting A-549 cell xenografts in the flank of nude mice. The treatments were initiated when the tumors reached ~70 mm^3^ (day 0) and ended 3 weeks later. (**A**) Normalized tumor volume during 6 weeks after initiating the three treatments: the conventional (unencapsulated) formulation of cisplatin and mifepristone, the drug-loaded liposomes (L-Cis/MF), and empty liposomes (the control group). (**B**) Final tumor volume after 6 weeks of initiating the treatments. (**C**) Body weight of the mice in the different groups during the 6 weeks after initiating the treatments. Data are expressed as the mean ± SEM (n = 6), and significant differences (*p* < 0.05) were examined by analysis of variance (ANOVA). * Significant difference between L-Cis/MF and the control; # Significant difference between the conventional formulation and the control.

An in vivo evaluation was made of the therapeutic efficacy of a 3-week treatment of tumor-bearing mice with the liposomal or conventional formulation of the cisplatin/mifepristone combination. The mice had received xenotransplants of A-549 cells of non-small-cell lung cancer (Figure 7). During the 6 weeks after initiating the treatments, tumor growth was better controlled by L-Cis/MF than the unencapsulated cisplatin/mifepristone combination. In relation to the initial tumor volume, the increase found was around 21-fold for the control group, 15-fold for the conventional formulation, and 7.5-fold for the liposomal nanosystem (Figure 7A,B). Regarding possible systemic toxicity, there was no significant change in animal body weight during the experiment and the general condition of the animals was normal (Figure 7C).

### 2.4. Quantitative Detection of VEGF in Tumors

**Figure 8:**
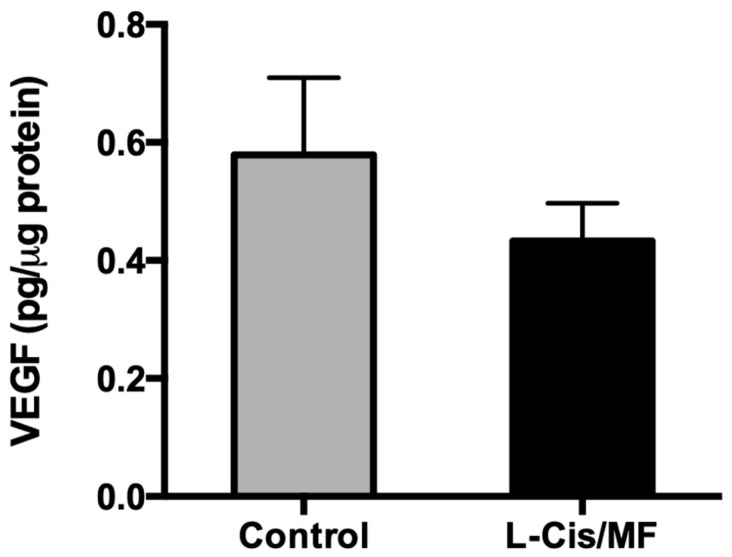
At the end of the study, analysis of the tissue of whole lysed tumors (A-540 xenografts) evidenced a slightly but not significantly lower level of the VEGF protein for the L-Cis/MF versus vehicle (control) treatment group.

Analysis of the tissue of the xenografts of non-small-cell lung cancer at the end of the study showed a slightly but not significantly lower protein level of VEGF for the L-Cis/MF treatment versus the control (Figure 8).

## 3. Discussion

### 3.1. Strategies for Cancer Therapy with Cisplatin

Cancer is characterized by unspecific symptomology during the first stages of development. Consequently, the majority of patients are diagnosed in advanced stages of the disease and treated with chemotherapy. Although antineoplastic treatments have improved significantly over the last decade, they still leave much to be desired. For example, cisplatin is a chemotherapy drug with a widespread clinical use for various types of cancer due to its effectiveness in eliminating tumor tissue. Nevertheless, chemotherapy with this drug is associated with severe adverse effects, including nephrotoxicity, neurotoxicity, ototoxicity, and myelotoxicity [1,2]. Moreover, it frequently leads to the development of tumor resistance, causing most patients treated with cisplatin to experience failure and relapse and therefore to have a poor prognosis. As a result of all the aforementioned factors, nowadays the standard treatment for the majority of advanced-stage cancer treated with cisplatin consists of a combination of drugs [17]. The combination of cisplatin with mifepristone has shown synergistic effects, but the problem of low bioavailability in tumor tissue remains [9,18].

Nanotechnology has been employed to develop drug delivery systems in order to achieve greater systemic distribution and bioavailability of drugs, leading to an improved selectivity and efficacy of therapy as well as reduced adverse effects. Various systems of drug delivery exist, including solid lipidic nanoparticles, polymeric micelles, and liposomes [19]. Recently, the co-delivery of antineoplastic drugs in a single nanosystem has been described [20,21,22]. In tests carried out with various formulations of nanoparticles containing a cytotoxic drug and a sensitizing agent, synergism has been found in the treatment of cancer [23].

Our group has reported the co-encapsulation of cisplatin and mifepristone by a nanosystem (L-Cis/MF) with a lipidic membrane made of HSPC, cholesterol, and DSPE-mPEG2000 [15]. In vitro assays have revealed the dynamics of drug release from the encapsulated formulation. Meanwhile, in vivo assays utilizing xenotransplants of cervical cancer have demonstrated that the liposomal co-encapsulation of cisplatin and mifepristone affords a significantly greater inhibition of tumor growth than the conventional (unencapsulated) formulation of the same combination [15]. Encapsulation is an interesting way to efficiently deposit both drugs at the tumor site. It achieves a better synergistic response than that observed with the conventional application of the same drugs.

Defining the pharmacokinetics of the L-Cis/MF nanosystem is an essential part of its preclinical characterization, as is the evaluation of the therapeutic efficacy of this nanosystem in distinct experimental animal models. One such model is the implantation of xenografts of non-small-cell lung cancer, for which cisplatin constitutes part of the standard treatment. These steps of preclinical characterization may facilitate clinical trials of the L-Cis/MF nanosystem in the future.

### 3.2. Profile of the Release of Cisplatin and Mifepristone from L-Cis/MF

The good stability of the present liposomal nanosystem in human plasma at 37 °C led to prolonged release of the two drugs. The percentage of release of cisplatin was 19% (Figure 2A). The current results correlate with previous publications describing a slow rate of release of hydrophilic chemotherapy agents co-encapsulated with sensitizing agents in liposomal systems [24,25].

Mendes et al. (2014) reported the co-encapsulation of genistein and paclitaxel in a multicompartmental nanostructured system. Genistein was entrapped in the external phospholipid bilayer of the polymeric nanoparticle and was released quickly (85% during the first 48 h). Paclitaxel, on the other hand, was encapsulated in the internal compartment of the polymeric core and showed a gradual and sustained release constituting only 10% of the compound [25]. A similar outcome was found with the present liposomal nanosystem. Cisplatin was encapsulated in the center of the formulation and was slowly and gradually released, leading to a low overall delivery.

Some studies on liposomal systems containing cisplatin have described elevated rates of the release of this drug (up to 50% at 24 h) [26,27,28]. Contrarily, the current formulation (L-Cis/MF) released 13% of the cisplatin in 24 h, possibly indicating the greater stability of the co-encapsulation of drugs in the same liposomal system. The slow release of cisplatin from the liposomal formulation could stem from various factors including the rigidity of the liposomal membrane and the physicochemical characteristics of the encapsulated drug. The mechanisms of release may involve transport by means of the polymer and/or the dissolution of the polymeric capsule. Accordingly, the release of the drugs encapsulated in the core of the liposomal formulation can be explained by slowly occurring processes, such as diffusion, hydration of the polymers, swelling, and degradation [25].

In contrast, the external envelope (the phospholipid bilayer) undergoes a dynamic and more rapid process, accounting for the more rapid release of mifepristone than cisplatin from L-Cis/MF, reaching 70% at 72 h (Figure 2B). Mifepristone is characterized by high lipophilicity, which favors its location in the external lipidic bilayer.

The location of drugs within the phospholipid membranes depends on their solubility as well as the hydrogen bonds or Van der Waals interactions between a hydrophobic drug and the hydrophobic chains of phospholipids in the bilayer. The composition of the lipidic membrane and the lipophilicity of the drug strongly influence the dynamics of drug release [29]. Hence, the percentage of release of mifepristone from the L-Cis/MF formulation is likely due in part to the dynamics of the lipids in the membrane [30,31].

### 3.3. Pharmacokinetics of L-Cis/MF

The pharmacokinetics of the release of cisplatin into the bloodstream was evaluated for the liposomal and conventional formulations. When comparing the administration of L-Cis/MF and free cisplatin, the former led to a higher plasma concentration of cisplatin over time, resulting in a significantly higher AUC, a lower rate of clearance, and a smaller volume of distribution (Figure 3, Table 1). In the liposomal formulation, as can be appreciated, there was a substantial increase in bioavailability and a slower rate of elimination of cisplatin, as well as a more limited distribution in tissues (probably greater specificity for tumor tissue). This may be due to the modification of the liposomal system by attaching polyethylene glycol (PEG)-units to the bilayer, leading to pegylated (stealth) liposomes. The latter prolongs systemic circulation in the bloodstream through a reduction in recognition by the mononuclear phagocytic system, and also through a decrease in interaction with plasma opsonins. As a consequence, stealth liposomes enhance the stability of encapsulated drugs, diminish their toxicity, increase their half-life, and decrease their clearance and immunogenicity [32,33].

Only a small percentage of conventional antineoplastic drugs in systemic circulation reach tumor tissue. These drugs leave the intravascular space by crossing the capillary wall to the interstitium through a process called extravasation. Subsequently, the free drugs are taken up by tumor cells by means of passive diffusion [34,35]. In contrast, PEGylated liposomes are usually internalized by endocytosis. They have the potential for improved drug targeting, and for controlled release from the nanosystem by passive targeting through a phenomenon known as the enhanced permeability and retention (EPR) effect in solid tumors. Due to the difference in vascular structure between tumor tissue and normal tissue, and to the nanoparticle size, the EPR effect favors a greater accumulation of liposomes in tumor cells [36].

When the tumor reaches a certain size, the original vessels are insufficient to supply the necessary oxygen and nutrients. Thus, cancer cells begin to develop a necrotic core, leading to the secretion of growth factors that trigger cell proliferation and angiogenesis, which in turn facilitate the rapid development of new blood vessels. Since these vessels have a discontinuous epithelium and lack a basement membrane, they contain spaces called fenestrae between endothelial cells, which makes it easier for the liposomes to reach tumor tissue [31].

Several studies have demonstrated that the pharmacokinetic profile of encapsulated drugs is modified significantly by the use of PEGylated liposomes [32]. The present results correlate with the data reported in the literature for liposomal formulations utilized clinically, such as PEGylated liposomal doxorubicin, commonly administered for the treatment of breast cancer. There is a significantly different pharmacokinetic profile for doxorubicin (a higher plasma concentration) with the application of the PEGylated liposomal versus the conventional (unencapsulated) formulation [37]. PEGylation offers a great advantage for anticancer drugs by decreasing immunogenicity, while increasing the time of systemic circulation and the serum half-life.

The in vitro assays evidenced a more rapid release of mifepristone than cisplatin, which may influence its rate of distribution and consequently its pharmacokinetic parameters. The pharmacokinetic evaluation showed a plasma concentration of mifepristone of 7.4 μg/mL following treatment with L-Cis/MF, with an AUC of 2.03 μg·h/mL·kg and a Vd of 939.82 mL/kg. The undetectable plasma level of mifepristone after administering the conventional formulation did not allow for the determination of the pharmacokinetic parameters.

Compared to the current results, a significantly lower plasma concentration of mifepristone was found in a previous study after rats were orally administered 10 mg/kg of mifepristone. The maximum plasma concentration was 0.167 μg/mL, with an AUC of 0.338 μg·h/mL·kg and a Vd of 1470 mL/kg [38]. This suggests that the use of liposome-based vectors to deliver drugs significantly modifies the pharmacokinetics of the encapsulated active agents, causing an increased plasma concentration and bioavailability of the drug, thus favoring a higher concentration in tumor tissue. Another relevant factor capable of affecting the efficacy of mifepristone treatment is its low solubility. An important challenge for many insoluble drugs, or those with limited solubility, is the development of new nanoformulations with the aim of enhancing their pharmacological potential.

### 3.4. The Therapeutic Efficacy of L-Cis/MF In Vivo

The therapeutic response, herein evaluated as the inhibition of tumor growth, was significantly better in the animals that received the liposomal versus the conventional (unencapsulated) formulation of the cisplatin/mifepristone combination. A previous report from our group demonstrated significantly greater drug accumulation in tumor xenografts of the cervix after the injection of a liposomal nanosystem containing cisplatin compared to the injection of unencapsulated free cisplatin [39]. The present comparison of the liposomal versus the conventional formulation reveals that the enhanced therapeutic response observed with L-Cis/MF correlates well with the increased bioavailability of the same (greater plasma concentration over time).

Overall, the better specificity of the liposomal formulation afforded superior cytotoxicity in tumor tissue as well as an absence of systemic toxicity (judging by the stability of animal weight). The pharmacokinetic characterization showed a significantly higher plasma concentration of both drugs in the animals that received L-Cis/MF versus the conventional (unencapsulated) formulation. Moreover, the distribution and elimination parameters were strongly modified, indicating a longer circulation time for both drugs. There was probably a higher concentration of the drugs in the tumor tissue. The hypothesis is that the co-encapsulated drugs were able to reach the tumor tissue more efficiently and accumulate there at a higher concentration than free drugs, resulting in a greater decrease in tumor growth. Further research on tissue distribution is needed to clearly define the mechanisms responsible for the inhibition of tumor growth stemming from treatment with L-Cis/MF.

Since the application of two or more drugs is a common practice in cancer treatment to achieve synergistic effects (and given our data about the better pharmacokinetics and greater efficacy of cisplatin and mifepristone co-loaded in liposomes), our work shows great prospects for designing new and better nanosystems of co-encapsulation, functionalized or conjugated with a variety of antibodies or ligands to target cancer cell receptors, enhancing drug delivery and internalization into tumor cells, and reducing drug distribution in normal tissue, so as to improve treatment outcomes in cancer therapy. The natural future step would be clinical trials on patients with cancer.

## 4. Materials and Methods

### 4.1. Drugs and Reagents

Cisplatin, mifepristone, trypsin, sodium chloride, nickel chloride, and sodium diethyl dithiocarbamate (DDTC) were supplied by Sigma–Aldrich Chemical Co. (St. Louis, MO, USA). Hydrogenated soybean L-α-phosphatidylcholine (HSPC), 1,2-distearoyl-sn-glycero-3-phosphoethanolamine-N-[methoxy(polyethyleneglycol)-2000] (DSPEm-PEG2000), and cholesterol were purchased from Avanti Polar Lipids (Birmingham, AL, USA). High-performance liquid chromatography (HPLC)-grade acetonitrile, chloroform, and methanol were acquired from Honeywell International, Inc. (Morristown, NJ, USA). Dulbecco’s modified Eagle’s medium (DMEM, Thermo Fisher Scientific Inc., Waltham, MA, USA), fetal calf serum (FCS, Thermo Fisher Scientific Inc., Waltham, MA, USA), ethylenediaminetetraacetic acid (EDTA, Thermo Fisher Scientific Inc., Waltham, MA, USA), Tris, and SDS were procured from GIBCO Inc. (Grand Island Biological Company, New York, NY, USA). High-quality water employed to prepare solutions was obtained with a Continental Milli-Q Reagent Water System (Millipore, El Paso, TX, USA).

### 4.2. Animals

Male Wistar rats (230–250 g, 6–7 weeks old) and male athymic Balb-C nu/nu mice (6–7 weeks old) were supplied by the Metropolitan Autonomous University (Universidad Autonoma Metropolitana, or UAM), Mexico City, Mexico. All animals were kept in a pathogen-free environment on a 12/12 h light/dark cycle and provided food and water ad libitum. Procedures for the care and handling of the animals were reviewed and approved by the Ethics Committee of the National Cancer Institute, Mexico City, Mexico (2020, with Ref. # 010/015/IBI-CB/619/10), and were in accordance with the Mexican Federal Regulation for Animal Experimentation and Care (NOM-062-ZOO-1999, Ministry of Agriculture, Mexico City, Mexico).

### 4.3. Preparation of Liposomes Co-Loaded with Cisplatin and Mifepristone

The liposomal formulation that co-encapsulated cisplatin and mifepristone (L-Cis/MF) was elaborated as described in Ledezma–Gallegos et al. (2020) [15]. Briefly, three lipids (HSPC, cholesterol, and DSPE-mPEG2000 at a molar ratio of 60:35:5) were dissolved in chloroform/methanol (2:1 *v*/*v*) and mixed with 5 mg of mifepristone. This mixture was deposited dropwise into a saturated cisplatin solution in sterile water (8 mg/mL) heated at 65 °C, maintaining a molar ratio of 1:12 for cisplatin and the phospholipids. The organic solvents were removed in a rotatory evaporator and the suspension was sonicated for 2 h to reduce and homogenize the size of the liposomes. Unencapsulated cisplatin was removed by dialysis using a 12,000 Da MWCO membrane (Spectrum Labs Inc., San Francisco, CA, USA), and unencapsulated mifepristone was removed by molecular exclusion chromatography on PD-10 columns packed with Sephadex-G-25 resin (GE Healthcare Inc., Chicago, IL, USA). Empty liposomes were prepared by following the same methodology but without adding any drugs. The liposomes were stored at 4 °C and protected from light. The amount of cisplatin and mifepristone co-encapsulated in the liposomes was measured, the former with HPLC based on the method described by our group [39], and the latter with another reported method [40].

Briefly, an aliquot of liposomes was transferred to a centrifuge tube, with promegestone serving as the internal standard (IS). After adding acetonitrile to disrupt the liposome and release the encapsulated mifepristone, the solution was centrifuged at 10,000 rpm and 4 °C. The supernatant was dried under an N_2_ atmosphere and resuspended in the mobile phase (water/acetonitrile), and then the sample was injected into the HPLC system. The mobile phase was a water/acetonitrile mixture delivered at 0.8 mL/min, and the detection system was set at 302 nm.

### 4.4. In Vitro Assay to Evaluate the Release of Cisplatin and Mifepristone from L-Cis/MF

The release of cisplatin and mifepristone from L-Cis/MF was assessed by using Franz diffusion cells assembled with a polycarbonate membrane (with 0.05 μm pores) (Millipore Corporation, Burlington, MA, USA) at 37 °C in human serum. An aliquot of L-Cis/MF was placed into the donor cell compartment (1 mL) and tamped down to the polycarbonate membrane. At specific time intervals (1, 2, 4, 8, 12, 24, 48, 72, and 96 h), the whole receptor phase medium (5 mL) was removed and replaced with an equal volume of fresh medium. The receptor phase was a saline solution for cisplatin and 2% sodium lauryl sulfate for mifepristone. The amount of cisplatin and mifepristone released was determined by the aforementioned HPLC methods.

### 4.5. Pharmacokinetics of L-Cis/MF

Pharmacokinetic studies were conducted on male Wistar rats anesthetized with isoflurane (Baxter, Mexico City, Mexico). The jugular vein was cannulated for the intravenous (IV) injection of the drugs, and the caudal artery was cannulated for blood sampling. Animals were randomly divided into three groups (n = 6) and given one of three treatments: (a) free cisplatin (6 mg/kg, IV); (b) free mifepristone administered subcutaneously (SC) (6.17 mg/kg); or (c) cisplatin/mifepristone-loaded liposomes (L-Cis/MF) applied IV at the same doses. Blood samples (300 µL) were collected from the caudal artery at 0, 5, 15, and 30 min and at 1, 2, 4, 6, 8, 24, 48, 72, and 96 h.

The concentration of cisplatin and mifepristone in plasma was evaluated by HPLC as aforementioned. The plasma samples were handled as detailed hereafter. The concentration of free cisplatin was determined in plasma that was ultra-filtered at 4 °C through Amicon Centriflo cones (10,000 molecular weight cut-off) immediately after drawing the blood sample. To quantify liposomal cisplatin, the methodology described by Toro-Córdova et al. (2016) was used [39]. Briefly, acetonitrile was added to 100 µL of plasma, and the samples were vortexed and centrifuged at 10,000 rpm for for 10 min. The supernatant was transferred to an Eppendorf tube and dried under N2 atmosphere. The pellet was suspended in 0.9% sodium chloride; nickel chloride was used as internal standard. After the samples were derivatized with DDTC in NaOH and incubated at 37 °C for 30 min, cisplatin was extracted with 100 μL of chloroform. Finally, 20 μL of the chloroform layer was injected into the chromatographic system, which consisted of a Waters 650E solvent delivery (Waters Assoc., Milford, MA, USA), a 20-μL loop injector (Rheodyne, Cotati, CA, USA) and a UV detector 486. Separation was carried out at 23 °C on a 150 × 3.9 mm ID Symmetry C18 column of 4 μm particle size. The mobile phase was a mixture of water, methanol, and acetonitrile. Detection was performed at 254 nm.

To quantify mifepristone, acetonitrile was added to an aliquot of plasma, and the mixture was centrifuged at 10,000 rpm. The resulting supernatant was dried and resuspended, and mifepristone was isolated with Oasis-HLB 3cc columns (Waters, Milford, MA, USA). The amount of mifepristone was established by utilizing the aforementioned method.

Plasma concentrations of cisplatin and mifepristone were plotted against time, and the following pharmacokinetic parameters were obtained on WinNonlin**^®^** Proffesional Edition Version 2.0 software (Certara, Princeton, NJ, USA): the area under the concentration-time curve (AUC), the elimination half-life (t_1/2_), clearance (Cl), the volume of distribution (Vd), and the plasma concentration at time zero (Cmax).

### 4.6. Tumor Xenografts and Systemic Toxicity

Athymic (nu/nu) male nude mice (6–7 weeks of age) were inoculated SC in the back with 5 × 10^6^ A549 (ATCC**^®^** CCL185TM) cells, a model for non-small-cell lung cancer. Tumor growth was monitored weekly by measuring two perpendicular diameters with a caliper. Tumor volume was calculated with the following equation: V = π/6 × (large diameter × [small diameter]/2). Once the tumor volume reached approximately 70 mm^3^, the animals were randomly divided into three groups (n = 6) to initiate treatment: (1) empty liposomes (L-Control); (2) free cisplatin (5 mg/kg/week) plus free mifepristone (9.7 mg/kg/week); and (3) liposomes loaded with cisplatin (at 5 mg/kg/week) and mifepristone (at 9.7 mg/kg/week) (L-Cis/MF). All treatments were administered for three cycles (3 weeks), with an intraperitoneal injection of all drugs, except for an SC injection of free mifepristone.

A weekly evaluation was made (for 6 weeks as of the beginning of the treatments) of the therapeutic efficacy and systemic toxicity by determining the tumor volume and recording animal weight (as well as by observing the general condition of the animals). The growth rate of tumors was analyzed with a graph by plotting normalized tumor volume against the number of days after initiating treatment. Normalized tumor volume (VNi) was calculated as VNi = Vi/V0, where Vi is the volume on a specific day i, and V0 is its initial volume on day 0 [41]. Systemic toxicity was considered at 20% of weight loss. At the end of the experiment, the mice were euthanized under anesthesia (isoflurane/oxygen 3%).

### 4.7. Quantitative Determination of VEGF in Tumors

The level of vascular endothelial growth factor (VEGF) in the tissue of the xenotransplants of non-small-cell lung cancer was assessed at the end of the study with an ultra-sensitive ELISA kit (ENZO Life Sciences, Farmingdale, NY, USA). Briefly, whole tumors were lysed, and the total protein content was isolated. Samples of each treatment were assayed according to the manufacturer’s protocol. Optical density was measured at a wavelength of 450 nm on a Multiskan Microplate Reader (Thermo Fisher Scientific, Waltham, MA, USA). The results, calculated by using a calibration curve, were expressed as pg VEGF/g of tissue.

### 4.8. Statistical Analysis

Data are expressed as the mean ± standard error of the mean (SEM). Significant differences between groups (considered at *p* < 0.05) were established by one-way analysis of variance (ANOVA) followed by the Bonferroni test. These tests were processed on SPSS 20.0 software (SPSS Inc., Chicago, IL, USA).

## 5. Conclusions

Novel anti-cancer therapeutic strategies now exist based on nanomedicine. They represent a promising approach due to their capability for overcoming the lack of specificity in conventional chemotherapeutic delivery systems, a problem responsible for an inadequate concentration of drugs at the tumor site and toxicity for normal cells. To increase the pharmacological effect through synergism, nanoparticle delivery systems can be loaded with two drugs possessing different mechanisms of action, such as the present case of combining a chemotherapy agent (cisplatin) with a sensitizing agent (mifepristone) that counters the drug resistance mechanism of tumors. The co-encapsulation of these two drugs in a liposomal nanosystem (L-Cis/MF) led to better pharmacokinetics (i.e., a higher concentration in blood plasma over a longer period of time), correlating with greater efficacy in the control of the growth of tumors resulting from the growth of xenografts of non-small-cell lung cancer. In future research, in vitro and in vivo assays will be used to characterize the molecular mechanisms of L-Cis/MF. Additionally, this liposomal nanosystem will be applied to other types of cancer that are likely to be affected by the synergism of cisplatin and mifepristone.

## Figures and Tables

**Table 1:** Pharmacokinetic parameters of cisplatin after intravenous administration of conventional cisplatin (unencapsulated) or cisplatin-loaded liposomes (L-Cis/MF).

Parameter	Conventional Cisplatin	Liposomal Cisplatin
AUC_0→t_ (μg·h/mL·kg)	5.27 ± 0.66	801.76 ± 77.88 *
t_1⁄2_ (h)	5.73 ± 2.25	17.26 ± 3.73 *
C_max_ (μg/mL)	9.62 ± 0.66	61.17 ± 3.07 *
Vd (mL/kg)	636.73 ± 39.64	169.11 ± 16.35 *
Cl (mL/h)	1286.53 ± 248.37	7.95 ± 0.76 *

(*) Significant difference (*p* < 0.05) between conventional cisplatin and liposomal cisplatin (L-Cis/MF) based on Student’s *t*-test. AUC, area under the curve; t_1/2_, the elimination half-life; Cmax, plasma concentration at time zero; Vd, volume of distribution; and Cl, clearance. Values are expressed as the mean ± SEM (n = 6).

**Table 2:** Pharmacokinetic parameters of free mifepristone or mifepristone after the intravenous administration of L-Cis/MF.

Parameter	Conventional Mifepristone	Liposomal Mifepristone
AUC_0→t_ (μg·h/mL·kg)	Not detectable	2.03 ± 0.26
t_1⁄2_ (h)	Not detectable	0.4 ± 0.16
C_max_ (μg/mL)	Not detectable	7.4 ± 1.01
Vd (mL/kg)	Not detectable	939.82 ± 105.40
Cl (mL/h)	Not detectable	3290.54 ± 351.22

AUC, area under the curve; t_1/2_, the elimination half-life; Cmax, plasma concentration at time zero; Vd, volume of distribution; and Cl, clearance. Values are expressed as the mean ± SEM (n = 6).

## Data Availability

Data is contained within the article.
